# Efficacy of canine-guided and bilateral balanced occlusion appliances in managing TMJ disc displacement with Reduction-A randomised clinical trial

**DOI:** 10.1016/j.jobcr.2026.101412

**Published:** 2026-01-31

**Authors:** Renita Lorina Castelino, Chethan Hegde, Srikant Natarajan

**Affiliations:** a Nitte (Deemed to be University), A.B.Shetty Memorial Institute of Dental Sciences, Department of Oral Medicine and Radiology, Mangalore, India; b Nitte (Deemed to be University), A.B.Shetty Memorial Institute of Dental Sciences, Department of Prosthodontics and Crown & Bridge, Mangalore, India; c Department of Oral Pathology and Microbiology, Manipal College of Dental Sciences Mangalore, India; d Manipal Academy of Higher Education, Manipal, India

**Keywords:** Health, Quality of life, Temporomandibular joint, Appliance, Orthotic

## Abstract

**Background:**

Among the intracapsular disorders of the temporomandibular joint (TMJ), disc displacement with reduction (DDwR) occurs most frequently. Canine-guided orthotic appliances may increase loading on the contralateral TMJ during eccentric movements due to the leverage effect generated on the working side whereas bilateral balanced occlusion appliances may promote uniform force distribution, reducing contralateral joint stress in patients with DDwR. Evidence comparing the effectiveness of canine-guided and bilateral balanced occlusion orthotic appliances in the management of DDwR remains limited.

**Aim:**

This study compared the effectiveness of canine-guided and bilateral balanced occlusion orthotic appliances in reducing pain, assessed using the Visual Analog Scale (VAS), in patients with DDwR.

**Material and methods:**

This study included 68 patients with painful TMJ DDwR (RDC/TMD Group IIa). Participants were randomly assigned to two groups: Group 1 received a canine-guided orthotic appliance, and Group 2 received a bilateral balanced occlusion orthotic appliance. Pain intensity was assessed using the VAS at baseline, 15, 30, and 90 days.

**Results:**

Mann-Whitney *U* test was used for group comparisons which demonstrated Group 2 achieved significantly lower VAS scores during early assessments, while Group 1 exhibited more notable reductions between 30 and 90 days. Wilcoxon test was used to assess improvement within groups which demonstrated significant improvement in both the groups with p value < 0.001.

**Conclusions:**

The bilateral balanced occlusion appliance produced faster pain reduction with lower VAS scores, particularly between 15 and 30 days. The canine-guided appliance showed greater pain reduction between 30 and 90 days. Both interventions effectively reduced pain.

## 1 Introduction

The American Academy of Orofacial Pain defines Temporomandibular Joint Disorders (TMD's) as “a group of disorders which involves the masticatory muscles, the TMJ, and the associated structures”.^1^ TMD pain is the most prevalent type of non-odontogenic pain contributing significantly to healthcare expenditure, treatment demand and deterioration in quality of life.^2^ Among TMD’S, pain-related conditions and intra-articular abnormalities are the most commonly observed.^3^ Intra-articular pathologies of the TMJ refer to changes that disturb the normal alignment between the condyle, disc, articular eminence and the fossa.^4^ Epidemiological studies show that DDwR accounts for about 41 % of all TMD cases, underscoring its importance as a common intra-articular TMJ pathology.^5^ It has been established that 33 % of cases of DDwR are asymptomatic in nature^6^ as TMJ structures adapt well to the abnormal disc position. In cases of DDwR where symptoms are present, the pain is generally believed to result mainly from inflammation within the joint.^7^ DDwR is characterized by disc displacement in the closed-mouth position, followed by recapture of the disc during mouth opening typically producing an audible click.^7^ Many conservative treatment options like patient education, relaxation techniques, exercises and the use of full coverage orthotic stabilization appliances^8^ are available to treat symptomatic cases of DDwR. For symptomatic cases of DDwR, the canine-guided orthotic stabilization appliance is frequently employed as a therapeutic option. Several studies have demonstrated that canine guidance contributes significantly to the functional success of orthotic appliances and the preservation of natural occlusal dynamics.^8^

Contact in canine guidance immediately causes reduction in the activity of the temporalis and masseter muscles, thereby decreasing extent of occlusal force as described by D'Amico in his reserach^9^ but may increase the loading on the non-working side of the TMJ during eccentric movements.^8^ Researchers have suggested that nonworking-side occlusal contacts may provide therapeutic benefits in reducing symptoms of painful TMJ disorders,^10^ a finding further supported by Kahan et al.^11^ Studies have shown that the mechanoreceptors present in the periodontal ligaments of the canines can modulate elevator muscle contraction.^8^ Studies have shown that during unilateral clenching, the load transmitted to the opposite or counter-lateral TMJ increases because of the leverage effect created by canine guidance on the working side.^8^ Introducing contact on the nonworking side can provide greater joint stability and helps reduce overall joint loading. This reduction in stress may support the healing process, leading to faster recovery in individuals experiencing TMJ pain.^8^ Bilateral balanced occlusion orthotic appliances achieves early load redistribution, whereas canine guidance orthotic appliances relies on gradual neuromuscular adaptation for its therapeutic effect.

Balanced occlusion appliances in centric occlusion are widely used in the management of TMDs; however, excursive balanced occlusion has not been commonly employed, and its application specifically in cases of DDwR remains limited. Hence, this research was undertaken to evaluate and compare the effectiveness of canine-guided and bilateral balanced occlusion orthotic appliances in alleviating pain associated with DDwR.

The null hypothesis H_0_ proposed that there is no significant difference in pain reduction, assessed using the VAS, at predefined follow-up intervals between patients with DDwR treated with canine-guided and bilateral balanced occlusion orthotic appliances. In contrast, the alternative hypothesis H_1_ stated that there is a significant difference in pain reduction, assessed using the VAS, at predefined follow-up intervals between patients with DDwR treated with canine-guided and bilateral balanced occlusion orthotic appliances.

From a clinical standpoint, the choice of appliance presents a clinical decision dilemma between achieving prompt pain relief and supporting sustained improvement over time.

## 2 Materials and Methods

This randomized clinical trial was designed as a prospective, parallel-group, single blinded study with a 1:1 allocation ratio, comparing the efficacy of canine-guided and balanced occlusal appliances in patients with DDwR. All procedures adhered to the CONSORT 2022 guidelines and the ethical principles of the Declaration of Helsinki (1975, revised 2013).

The study protocol received approval from the Central Ethics Committee (Approval No. NU/CEC/2024/531). The trial was registered prospectively with the Clinical Trials Registry of India (CTRI/2024/04/065372), and participants were enrolled according to predefined criteria. All participants provided written informed consent.

68 patients aged between 18 and 40 years, presenting with clinical signs and symptoms of DDwR associated with pain and clicking of the joint, belonging to RDC/TMD Group IIa, and who were willing to participate, were included in the study. Participants included in groups other than Group IIa, and with systemic joint disorders, a prior history of temporomandibular joint surgery, or demonstrated non-compliance with appliance usage were excluded from the study.

All patients were recruited from the outpatient department after undergoing standard diagnostic investigations and evaluations. Patient history was systematically recorded using an approved questionnaire to ensure standardized assessment. A comprehensive intraoral and extraoral examination was also performed. The diagnosis of DDwR was established using standardized clinical criteria as proposed by Dworkin and LeResche.^12^ According to these criteria, diagnosis was based on the “presence of reciprocal clicking in the TMJ (click on both opening and closing occurring at least 5 mm greater interincisal distance on opening than on closing, and eliminated on protrusive opening), reproducible on two of three consecutive trials, or clicking detected during both vertical mandibular movements along with clicking on protrusion or lateral excursion or which are reproducible on two of three trials”.

Participants were randomized to receive either a canine-guided or a balanced occlusal appliance. The fabrication of the orthotic appliances was done as outlined by Okeson.^13^

The patient was seated upright, and an appropriately sized dentate impression tray was selected. An irreversible hydrocolloid (Zhermack Neocolloid, Italy)was mixed as instructed by manufacturer to a creamy consistency and used to record the maxillary impression, which was then rinsed under running water. A working cast was fabricated using Type II dental stone (Goldstone, India). A 1.5 mm thick, clear acrylic hard resin (Bio-Art Dental Lab Splint thermoforming material, Brazil) was adapted to the cast utilising a vacuum adapter (Termoformadora Sabilex). Keeping the height of the interdental papilla on the labial and buccal aspects of the teeth at around 10–12 mm from the gingival border of teeth on the lingual side, the outline of the orthotic appliance was designed. The labial margin of the appliance was positioned between the middle and incisal thirds of the anterior teeth. A dis-occluding ramp of dimension around 2 mm in width and 6 mm in length was prepared using auto polymerizing resin (DPI RR Cold cure, India) along the palatal contour behind the upper central incisors 11 and 21 to deprogram the muscles. The appliance was tried in the patient's mouth to ensure around 3 mm clearance between the upper and lower anterior teeth. Muscle deprogramming was done by making the patient wear this appliance for a minimum of 15 min to break the effect of any possible engrams while recording centric relation position. An auto polymerizing acrylic resin (DPI RR Cold cure, India) was mixed as instructed by the manufacture and adapted along the maxillary dental arch on the appliance. The centric relation record was obtained using Dawson's bimanual method, whereby the clinician gently supports and guides the mandible into its optimal condylar position, minimizing the influence of muscle memory or habitual occlusion. While the material was setting, the patient was guided to open and close the mouth to prevent locking of mandibular teeth into the resin. Once the material set, and after the desired centric contacts were achieved, refinement of the anterior guidance was done to allow the mandibular canines to move in a smooth and continuous manner during any eccentric movements. The excessive acrylic resin over the mandibular buccal and lingual cusps were trimmed. Canine guidance should allow smooth and gentle dis-occlusion of the posterior teeth. Articulating paper was used to identify any additional contacts in eccentric movements, which were subsequently eliminated. The design enabled posterior disocclusion, guided by canines in lateral and anterior teeth in protrusive movements. Patients in Group 1 received this appliance design.

For the bilateral balanced occlusion appliance, the appliance was prepared similar to as explained above to achieve uniform contact of mandibular teeth against the maxillary orthotic appliance. In order to attain bilateral balanced occlusion, the patient was guided to right lateral, left lateral and protrusive movements. Any contacts recorded using the articulating paper causing disocclusion were eliminated to provide bilateral balanced occlusion. Acrylic resin was added to develop contacts in case of one or two teeth were unable to provide working and non -working contacts. This design ensured that the buccal cusps and incisal edges of the mandibular teeth contacted a flat surface, allowing even and simultaneous contact of all mandibular teeth during excursive movements. Patients in Group 2 received this appliance design.

After fabrication, the appliance was delivered to the patient, who was instructed to wear it during sleep and additionally for 4 h in the morning and 4 h in the evening. When not in use, patients were advised to store the appliance in water and clean it with a brush and mild soap. All appliances were fabricated and adjusted by a single operator to control for operator-related variability. Occlusal equilibration was performed only when clinically indicated based on appliance wear and tear, and the frequency of equilibration did not differ between the two groups. The primary outcome was the evaluation of pain intensity using a 10-cm Visual Analog Scale (VAS), where ‘0’ constituted absence of pain and ‘10’ the worst imaginable pain. Participants marked their perceived pain level on the scale, and the distance from the left end was recorded as the VAS score. All co-interventions, including medications, physiotherapy, and behavioural advice, were restricted during the study to minimise confounding effects. Assessments were performed at baseline and at predetermined follow-up intervals of 15, 30, and 90 days.

Participants were randomized using block randomization with a fixed block size of 4 to ensure balanced allocation between Group 1 and Group 2 throughout the enrolment period. The randomization sequence was generated by an independent investigator, who was not involved in participant recruitment, clinical intervention, or outcome assessment, using computer-based random numbers. Each participant's group assignment (‘A’ for control and ‘B’ for intervention) along with the participant number was recorded on a card, which was placed inside an opaque envelope which was sequentially numbered. Envelopes were unsealed only immediately prior to the intervention by the recruiting clinician, maintaining strict allocation concealment. Owing to the characteristics of the intervention, participants could not be blinded to the type of appliance, and the treating clinician was aware of group allocation. Blinding was maintained at the level of outcome assessment, making this a single-blind study.

Since no previous studies had assessed the effectiveness of an orthotic appliance with balanced occlusion for disc displacement with reduction, a pilot observational study was first conducted with five participants. The findings from this pilot study were then used to calculate the sample size. The calculations were made based on the following parameters: standard deviation in Group 1 was 0.8366 and in Group 2 was 0.8366, mean difference was 0.6, and effect size was 0.71718. With an alpha error of 5 % and a power of 80 % (two-sided test), the required sample size was determined to be 31 participants per group. After accounting for a 10 % attrition rate, the final sample size was increased to 34 participants in each group, yielding a total of 68 participants. The sample size calculation was performed using nMaster software version 2.

The data recorded was compiled using MS Office Excel (version 2019, Microsoft, Redmond, WA, USA). IBM Statistical Package for Social Sciences (SPSS) version 20 was used to analyse data. The normality of the data was assessed using the Shapiro–Wilk test which showed non-normal distribution (p < 0.05) for most of the variables and hence non-parametric statistical tests were used. Categorical variables were analysed using the Pearson Chi-square test. Differences between the two independent groups were evaluated using the Mann–Whitney *U* test. Intra-group changes were evaluated with the Wilcoxon signed-rank test for paired comparisons of VAS scores at different time intervals. A p-value of less than 0.05 was considered statistically significant.

## 3 Results

**Fig. 1:**
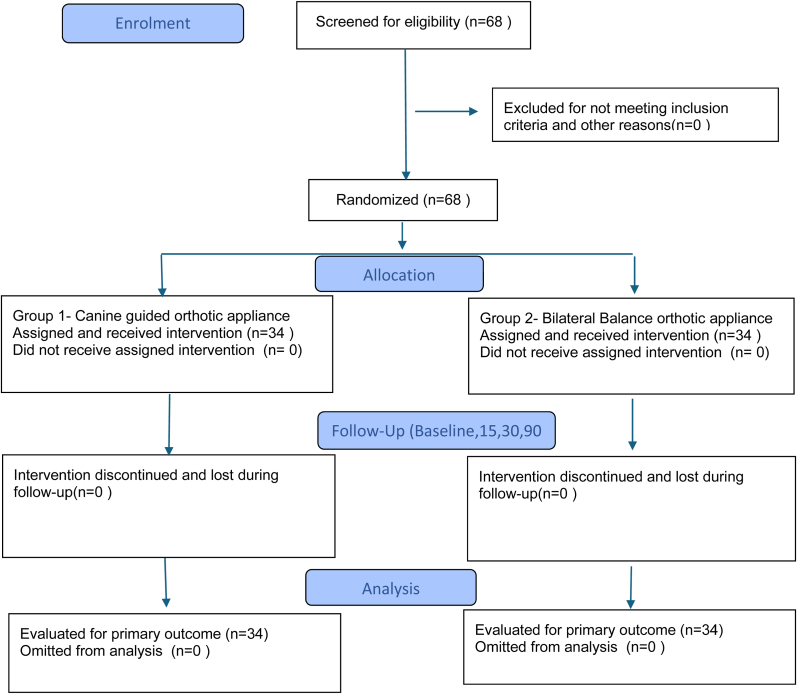
CONSORT flow Diagram.

A total of sixty eight patients were screened for eligibility, all of whom met the inclusion criteria and were randomized equally into two groups (n = 34 per group). All randomized patients completed the 90-day follow-up and were analysed in the final analysis. No participants were omitted from analysis. Participant flow is summarised in the CONSORT flow diagram (Fig. 1).

The gender distribution between the 2 groups was evaluated using the Chi-square test. In Group 1, females constituted 55.9 % (19/34) and males 44.1 % (15/34), while in Group 2, females accounted for 61.8 % (21/34) and males 38.2 % (13/34). Overall, females formed 58.8 % (40/68) and males 41.2 % (28/68) of the study population. Chi-square analysis revealed no statistically significant difference in gender distribution between the two groups (χ^2^ = 0.24, df = 1, p = 0.62), indicating that Group 1 and Group 2 were comparable with respect to gender composition (Table 1).

**Table 1:** Gender distribution between Group 1 and Group 2 using Chi Square test.

Gender	Group 1 n (%)	Group 2 n (%)	Total n (%)
Female	19 (55.9 %)	21 (61.8 %)	40 (58.8 %)
Male	15 (44.1 %)	13 (38.2 %)	28 (41.2 %)
Total	34 (100 %)	34 (100 %)	68 (100 %)

Chi-square = 0.24, df = 1, p = 0.62.

The two groups were similar at baseline with respect to age in years (Group 1: 29.38 ± 8.11 years; Group 2: 29.85 ± 8.43 years; p = 0.743) and maximum mouth opening (MMO) in mm (Group 1: 38.71 ± 7.53 mm; Group 2: 40.06 ± 5.43 mm; p = 0.376), with no statistically significant differences. (Table 2).

**Table 2:** Comparison of Baseline Characteristics (Age in years and Maximum Mouth Opening in mm) Between Group 1 and Group 2 using Mann Whitney *U* test.

		Count	Mean (sd)	Median (25th, 75th percentile)	Range	Sum of Ranks	Mann-Whitney U	P value
AGE	Group 1	34	29.38 (8.11)	28.5 (21, 37)	18 to 40	1146.5	551.5	0.743
	Group 2	34	29.85 (8.43)	30 (23, 40)	18 to 40	1199.5		
MMO	Group 1	34	38.71 (7.53)	39.5 (34, 42)	24 to 57	1101	506	0.376
	Group 2	34	40.06 (5.43)	38 (36, 43)	32 to 53	1245		

**Graph 1:**
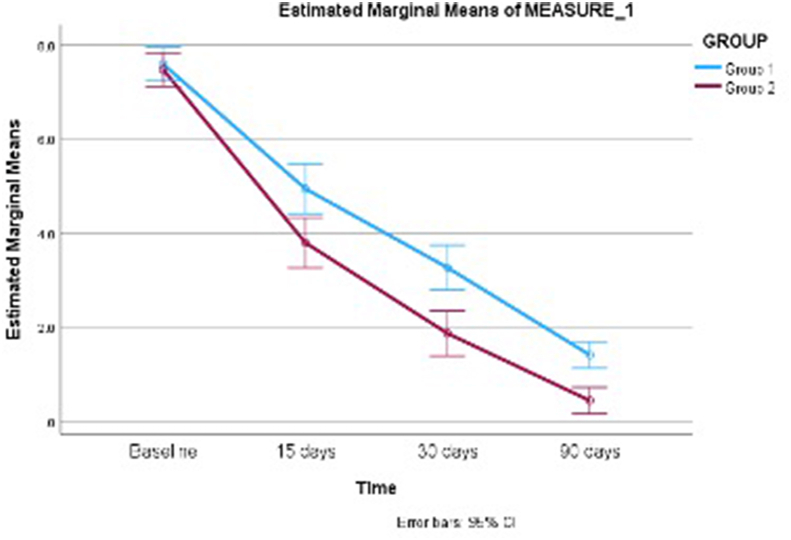
Line graph demonstrating the mean change in the values over time in Group 1 and 2 respectively.

Baseline VAS pain scores were also similar between the groups (Group 1: 7.59 ± 1.08; Group 2: 7.47 ± 1.02; U = 556; p = 0.779), confirming that participants began the study with comparable levels of pain. At 15 days, Group 1 reported significantly higher pain (4.94 ± 1.65) compared to Group 2 (3.79 ± 1.45; U = 319; p = 0.001), indicating greater early pain reduction in Group 2. This trend persisted at 30 days, with Group 1 demonstrating a mean pain score of 3.26 ± 1.31 and Group 2 a significantly lower score of 1.87 ± 1.48 (U = 250; p < 0.001). By 90 days, patients in Group 2 reported decrease in pain (0.44 ± 0.82) compared with Group I (1.41 ± 0.82), with the difference being statistically significant (U = 227.5; p < 0.001) as shown in Table 3, Graph 1.

**Table 3:** Comparison of Visual Analogue Scale (VAS) Pain Scores at Baseline and Follow-Up Intervals Between Group 1 and Group 2 using Mann Whitney *U* test.

		Count	Mean (sd)	Median (25th, 75th percentile)	Range	Sum of Ranks	Effect Size Rank biserial correlation	Mann-Whitney U	P value
Baseline	Group 1	34	7.59 (1.08)	7 (7, 8)	6 to 10	1195	−0.0381	556	0.779
	Group 2	34	7.47 (1.02)	8 (7, 8)	6 to 9	1151			
15 Days	Group 1	34	4.94 (1.65)	5 (4, 6)	0 to 7	1432	−0.4481	319	0.001
	Group 2	34	3.79 (1.45)	4 (3, 5)	1 to 7	914			
30 Days	Group 1	34	3.26 (1.31)	3 (3, 4)	0 to 5	1501	−0.5675	250	<0.001
	Group 2	34	1.87 (1.48)	2 (1, 2)	0 to 7	845			
90 Days	Group 1	34	1.41 (0.82)	1.5 (1, 2)	0 to 3	1523.5	−0.6064	227.5	<0.001
	Group 2	34	0.44 (0.82)	0 (0, 1)	0 to 3	822.5

The comparison of VAS pain scores between Group 1 and Group 2 at different follow-up intervals was evaluated using the Mann–Whitney *U* test, and the magnitude of differences was interpreted using the rank biserial correlation as the effect size. At baseline, the effect size was negligible (r = −0.0381), indicating virtually no difference in baseline pain scores between the two groups, which confirms baseline comparability. At 15 days, a moderate effect size was observed (r = −0.4481), reflecting a clinically meaningful reduction in pain scores in Group 2 compared to Group 1. This difference further increased at 30 days, showing a moderate-to-large effect size (r = −0.5675), and at 90 days, a large effect size was noted (r = −0.6064), indicating substantially lower pain scores in Group 2 at longer follow-up intervals (Table 3).

When changes from baseline were considered, the difference in pain reduction at 15 days demonstrated a large effect size (r = 0.5260), and at 30 days, the effect size remained large (r = 0.5433), suggesting that Group 2 experienced greater early pain reduction compared to Group 1. At 90 days, the effect size was moderate (r = 0.4092), indicating sustained but slightly reduced magnitude of difference in long-term pain reduction. In contrast, comparisons of pain reduction between intermediate time intervals showed minimal to small effects, with negligible effect size between 30 days and 15 days (r = 0.0657), a small effect between 90 days and 15 days (r = −0.1609), and a small-to-moderate effect between 90 days and 30 days (r = −0.3192), suggesting that most of the clinically relevant differences occurred early and were maintained rather than newly emerging at later intervals (Table 4).

**Table 4:** Comparison of Pain Reduction from Baseline Across Different Time Points Between Group 1 and Group 2 using Mann Whitney *U* test.

		Count	Mean (sd)	Median (25th, 75th percentile)	Range	Sum of Ranks	Effect Size Rank biserial correlation	Mann-Whitney U	P value
15 Days difference to baseline	Group 1	34	2.65 (1.74)	2 (2, 3)	1 to 10	869	0.5260	274	<0.001
	Group 2	34	3.68 (1.41)	3.5 (3, 4)	1 to 8	1477			
30 Days difference to baseline	Group 1	34	4.32 (1.17)	4 (4, 5)	2 to 8	859	0.5433	264	<0.001
	Group 2	34	5.6 (1.77)	6 (5, 7)	−1 to 9	1487			
90 Days difference to baseline	Group 1	34	6.18 (1)	6 (6, 7)	4 to 9	936.5	0.4092	341.5	0.003
	Group 2	34	7.03 (1.38)	7 (6, 8)	3 to 9	1409.5			
30 Days–15 days difference	Group 1	34	1.68 (1.34)	2 (1, 2)	−5 to 3	1135	0.0657	540	0.62
	Group 2	34	1.93 (1.54)	2 (1, 3)	−2 to 6	1211			
90 days–15 days difference	Group 1	34	3.53 (1.31)	4 (3, 4)	−2 to 5	1266	−0.1609	485	0.231
	Group 2	34	3.35 (1.5)	3 (2, 4)	0 to 7	1080			
90 days–30 days difference	Group 1	34	1.85 (0.66)	2 (2, 2)	0 to 3	1357.5	−0.3192	393.5	0.014
	Group 2	34	1.43 (0.87)	1 (1, 2)	0 to 4	988.5			

Group 2 ‘showed greater pain reduction from baseline at all time points (15 days: 3.68 ± 1.41 vs. 2.65 ± 1.74 U = 274, p < 0.001; 30 days: 5.60 ± 1.77 vs. 4.32 ± 1.17,U = 264, p < 0.001; 90 days: 7.03 ± 1.38 vs. 6.18 ± 1.00; U = 341.5, p = 0.003). No statistically significant differences existed between the groups for reductions from 15 to 30 days (U = 540,p = 0.620) or 15–90 days (U = 485,p = 0.231). From 30 to 90 days, Group 1 exhibited greater improvement (1.85 ± 0.66 vs. 1.43 ± 0.87; U = 393.5, p = 0.014), indicating notable pain relief in this period as depicted in (Table 4).

Table 5 shows intra group comparison of pain in Group 1 with mean VAS scores reduced from 7.59 ± 1.08 at baseline to 4.94 ± 1.65 at 15 days (Z = −5.188, p < 0.001), 3.26 ± 1.31 at 30 days (p < 0.001), and 1.41 ± 0.82 at 90 days (p < 0.001). Significant differences were also noted between 15 and 30 days (Z = −4.614, p < 0.001), 15–90 days (Z = −5.150, p < 0.001), and 30–90 days (Z = −5.206, p < 0.001).

**Table 5:** Intra group comparison VAS pain score changes in Group 1 using Wilcoxon Sign Rank test.

Comparison	Descriptive (Mean ± SD)	Median (IQR)	Range	Z value	p value
Baseline	7.59 ± 1.08	7 (7, 8)	6 to 10	–	–
15 Days	4.94 ± 1.65	5 (4, 6)	0 to 7	−5.188	<0.001
30 Days	3.26 ± 1.31	3 (3, 4)	0 to 5	−5.134	<0.001
90 Days	1.41 ± 0.82	1.5 (1, 2)	0 to 3	−5.173	<0.001
30 vs 15 Days	–	–	–	−4.614	<0.001
90 vs 15 Days	–	–	–	−5.150	<0.001
90 vs 30 Days	–	–	–	−5.206	<0.001

In Group 2, scores declined from 7.47 ± 1.02 at baseline to 3.79 ± 1.45 at 15 days (Z = −5.122, p < 0.001), 1.87 ± 1.48 at 30 days (p < 0.001), and 0.44 ± 0.82 at 90 days (p < 0.001), with significant reductions between 15 and 30 days (Z = −4.474, p < 0.001), 15–90 days (Z = −5.047, p < 0.001), and 30–90 days (Z = −4.893, p < 0.001) as depicted in Table 6.

**Table 6:** Intra group comparison VAS pain score changes in Group 2 using Wilcoxon Sign Rank test.

Comparison	Descriptive (Mean ± SD)	Median (IQR)	Range	Z value	p value
Baseline	7.47 ± 1.02	8 (7, 8)	6 to 9	–	–
15 Days	3.79 ± 1.45	4 (3, 5)	1 to 7	−5.122	<0.001
30 Days	1.87 ± 1.48	2 (1, 2)	0 to 7	−5.107	<0.001
90 Days	0.44 ± 0.82	0 (0, 1)	0 to 3	−5.121	<0.001
30 vs 15 Days	–	–	–	−4.474	<0.001
90 vs 15 Days	–	–	–	−5.047	<0.001
90 vs 30 Days	–	–	–	−4.893	<0.001

Both the groups showed statistically significant intra-group improvements in pain scores. Joint sounds were reduced in both the groups which was evaluated by clinical examination and patient reporting. Participants reported no changes in occlusion post the treatment.

## 4 Discussion

Orthotic appliances are commonly relied upon in everyday practice and are considered a trusted option for managing TMDs.^7^^,^^14^ Many types of orthotic appliances are available for treating various TMD's.^15^ The main function of an orthotic appliance is to protect the TMJ from any harmful stresses.^16^ The appliances usually redistribute the bite force over large area and thereby decrease the load on the joint. These appliances are known to alleviate pain, minimise further damage and thereby improve function which has been supported by various studies.^16^^,^^17^

TMDs are a group of conditions affecting the orofacial and dental structures, with a prevalence of about 20–40 % in the general population, often impacting health and overall quality of life. They are most commonly seen in individuals between 18 and 45 years of age and occur more frequently in females.^18^ DDwR is the most common form of intra capsular disorder of the TMJ characterised by progressive displacement of the disc against the condyle causing symptoms.^19^

Stabilization orthotic appliances are most commonly employed to treat TMJ pain or pain arising from the masticatory muscles.^20^^,^^21^ The various modalities used to treat DDwR are occlusal appliances, physical, behavioural, pharmacological or surgical therapies.^22^ These treatment options can be individually used or in combination.^23^ Inspite of various designs of the orthotic appliances available, stabilization appliances and anterior repositioning appliances are the most commonly used for the management of DDwR.^24^ Joint loading is influenced by the design of the orthotic appliance, as different occlusal schemes alter the way forces are distributed across the TMJ.^25^

Many studies have summed up that a part of the occlusal force applied to the teeth is transferred to the TMJ.^26^ Moreover, anterior the tooth contact on the appliance, greater is the load shifted to the joint.^27^ Consistent with previous reports, the TMJ is subjected to both distraction and compression forces during unilateral clenching.^28^ When concurrent contacts are set-up on both the working and nonworking sides, however, the stress on the opposite joint is markedly reduced.^29^ This supports the rationale for using a bilateral balanced occlusion appliance, as its design promotes even force distribution and may contribute to earlier symptom relief in patients with DDwR. Furthermore, given that individuals with disc displacement have a decreased capacity to absorb load occlusally, the use of balanced appliances could provide additional protection to the joint by minimizing excessive stresses.^30^

Reduced mouth opening is typically not a presenting feature in DDwR, especially when unaccompanied by muscle pain. Since our study aimed to evaluate the impact of orthotic therapy on symptomatic joints, only patients with painful joints were included, ensuring that the sample represented clinically significant cases.

The present study compared subjective pain reduction between two orthotic appliances for disc displacement using the VAS. This scale is widely regarded as a valid and reliable tool for pain assessment. It offers a simple, subjective measure that is easy for patients to understand and apply, making it suitable across diverse clinical settings.^31^ Given its widespread acceptance and frequent use in TMD research, the VAS was selected in the present trial to assess pain reduction in all participants, consistent with its use in previous studies.^8^^,^^31^ The reduction of the joint sounds can be attributed due to the remodelling of the associated structures rather than recapture of the disc, as suggested by Conti et al..^8^

In this study, the baseline VAS pain scores were comparable between the groups, indicating similar initial pain levels. In our study, participants were selected from the 18–40 year age group, which represents the age range most commonly affected by TMDs. Both groups were similar in terms of age. All participants demonstrated adequate mouth opening, with no significant differences observed between the two groups. Both Group 1 and Group 2 showed statistically significant improvements in VAS pain scores at each follow-up compared to baseline and the previous interval, confirming that both interventions effectively reduced pain. Baseline scores were comparable, allowing valid comparisons. Group 2 demonstrated greater pain reduction and faster recovery, particularly in the early phase, which may be attributed to enhanced joint stabilization due to even force distribution and improved occlusal balance, potentially resulting in reduced intra-articular pressure and TMJ loading. In contrast, Group 1 showed more gradual improvement between 30 and 90 days, reflecting recovery within the normal range of time and suggesting a progressive reduction in intra-articular pressure possibly due to altered load distribution. By 90 days, both groups achieved comparable recovery, indicating both interventions were effective.

Our results are consistent with earlier studies evaluating different therapeutic approaches for DDwR. Tahoon et al.^31^ reported significant pain reduction with both stabilization (canine guided) and anterior repositioning appliances. Conti et al.^8^ demonstrated that canine guidance and bilateral balanced splints achieved greater pain reduction than non-occluding appliances in cases of disc displacement. Similarly, Kurt et al.^24^ compared stabilization splints (canine guided), anterior repositioning splints, and behavioural therapy, and found that all groups showed improvement in cases of DDwR. A study by Al-Rafah EM et al.^32^ on patients with temporomandibular joint disorders, who were randomly assigned to receive either a canine-guided or bilateral balanced stabilization appliance, concluded that significant symptom improvement was observed.

Together, these findings reinforce the role of occlusal appliances in conservative DDwR management, with design features influencing the speed and degree of symptomatic improvement. The observed intra- and inter-group improvements contribute novel evidence to the literature, supporting the clinical effectiveness of both balanced occlusion and canine-guided orthotic appliances in managing disc displacement–related pain.

This is the first randomized clinical trial directly comparing canine-guided and bilateral balanced occlusion appliances specifically in DDwR. The key methodological strengths include prospective trial registration, ethical approval, and strict adherence to CONSORT guidelines. The absence of participant attrition and the achievement of complete follow-up also support the internal validity of the study.

This study has several limitations. The absence of MRI confirmation introduces the possibility of diagnostic misclassification, despite the established validity of RDC/TMD criteria. The study employed single blinding of outcome assessment, which may have influenced subjective pain reporting. The 90-day follow-up period limits evaluation of long-term stability and potential symptom recurrence. Functional outcomes, such as changes in mouth opening and quality of life, were not assessed despite their clinical relevance. Future studies should incorporate patient-centred outcome measures, including clicking severity, satisfaction, and quality-of-life assessments, to better capture patients’ perspectives on treatment efficacy and overall experience. Finally, the effectiveness of the appliance is influenced by patient compliance, which is challenging to monitor due to its home-based use, and the lack of direct supervision should be considered a potential limitation.

## 5 Conclusion

A significant and progressive reduction in pain was observed over time in both groups, demonstrating the effectiveness of each intervention. Both canine-guided and balanced occlusal appliances achieved clinically meaningful improvements in TMJ pain, reinforcing their value in the management of DDwR.

## Patient’s consent

A written informed consent was obtained from the all the patients enrolled in the study.

## Ethical clearance

This research was carried out after the approval obtained from the Central Ethics Committee (Approval No. NU/CEC/2024/531) and was also registered with the Clinical Trial Registry of India (CTRI/2024/04/065372).

## Contribution details

**Dr Renita Lorina Castelino:** Literature search, Data acquisition, Manuscript preparation, Manuscript review.

**Prof Dr Chethan Hegde**: Concepts, Design, Manuscript editing, Manuscript review.

**Prof Dr Srikant** **Natarajan**: Statistical data Analysis.

## Sources of funding

The research did not receive any funding and was carried out independently.

## Declaration of competing interest

The authors confirm that there are no conflicts of interest.
